# Characteristics of Patients Who Drop Out of Anti-Vascular Endothelial Growth Factor Therapy for Macular Edema Associated with Branch Retinal Vein Occlusion

**DOI:** 10.1155/2024/8336516

**Published:** 2024-07-05

**Authors:** Setsuko Kawakami, Yoshihiro Wakabayashi, Yoko Watanabe, Kazuhiko Umazume, Kaori Yamamoto, Hiroshi Goto

**Affiliations:** Department of Ophthalmology Tokyo Medical University, Tokyo 160-0023, Japan

## Abstract

**Purpose:**

To investigate the dropout rate of anti-vascular endothelial growth factor (VEGF) treatment for macular edema (ME) secondary to branch retinal vein occlusion (BRVO) and identify the characteristics of dropout cases.

**Methods:**

We studied 235 eyes of 235 treatment-naïve BRVO-ME patients receiving intravitreal injection of ranibizumab. Additional intravitreal anti-VEGF drug was given when ME relapsed or persisted, and photocoagulation was performed as needed. Adherence until treatment completion was defined as disappearance of ME within 2 years after the first injection without recurrence for more than 6 months or mild ME remaining but no visual deterioration for more than 6 months without additional anti-VEGF drug. In patients with ME recurrence, those who were followed for more than 2 years were considered adherence, and those followed for less than 2 years were considered dropout. The clinical course and background of the two groups were compared.

**Results:**

179 patients (76.2%) adhered to treatment and 56 patients (23.8%) dropped out. Mean follow-up periods in adherence and dropout groups were 23.4 and 7.1 months, respectively. There were no significant differences between the two groups in demographic and baseline factors of age, gender ratio, distance from home to hospital, visual acuity, and foveal thickness (FT). At the last follow-up, visual acuity was significantly poorer in the dropout group than in the adherence group (0.23 vs. 0.11 logMAR, *p*=0.003), and FT was significantly greater in the dropout group than in the adherence group (316 vs. 273 *µ*m, *p*=0.002). Reasons for dropout included patient declining further treatment in 12.5%, progression of dementia in 8.9%, others, and unknown in 64.3%.

**Conclusion:**

The clinical outcome of patients who dropped out of anti-VEGF therapy for BRVO-ME was worse compared to patients who adhered to therapy, and the reasons for discontinuation varied.

## 1. Introduction

Anti-vascular endothelial growth factor (VEGF) therapy is well known to be effective for macular edema (ME) associated with branch retinal vein occlusion (BRVO) [1, 2]. Although anti-VEGF therapy is also used for retinal diseases such as age-related macular degeneration (AMD) and diabetic macular edema (DME), patients are often treated with fewer injections in real-world clinical studies than in randomized clinical trials, resulting in inferior outcomes [3–6]. Patient adherence is considered critical to the success of therapy. However, some patients discontinue anti-VEGF therapy before their disease status stabilizes. Few reports have investigated the interruption of anti-VEGF therapy for macular edema associated with retinal vein occlusion (RVO-ME) [7–9]. Furthermore, even fewer reports distinguish between cases of completed or uncompleted therapy and investigate patient factors and outcome [7]. We investigated the proportion of patients treated with intravitreal injection of ranibizumab (IVR) injection for BRVO-ME who discontinued before completing treatment, i.e., dropped out of treatment, and the characteristics of these patients.

## 2. Materials and Methods

This retrospective case series consisted of 235 eyes of 235 consecutive patients with BRVO-ME, in whom IVR therapy was initiated at Tokyo Medical University Hospital between September 2013 and August 2018. The study adhered to the tenets of the Declaration of Helsinki and was approved by the institutional review board. All subjects provided written informed consent. In this study, we obtained informed consent for both anti-VEGF therapy for BRVO-ME and for the retrospective study using information from the medical records. Eyes with other retinal disorders and eyes with a history of intravitreal injection of anti-VEGF drugs, intravitreal or sub-Tenon's injection of triamcinolone acetonide (STTA), retinal photocoagulation, or vitreous surgery were excluded.

Before the first IVR injection and every month thereafter, all patients underwent ophthalmic examinations including best-corrected visual acuity (BCVA) measurement, slit-lamp biomicroscopy, fundus examination, and optical coherence tomography (OCT). When OCT showed relapse or persistent exudative changes after the first 0.5 mg ranibizumab injection, additional 0.5 mg ranibizumab injection was given as needed (pro re nata: PRN). When the effect of IVR injection was absent or diminished, or ME relapsed frequently, the drug was switched to 2 mg aflibercept or STTA was given in some cases as decided by the physician. After retinal hemorrhage was reduced, fluorescein angiography (FA) was performed. Scatter photocoagulation (PC) was done when non-perfusion area (NPA) was 5 papillary disc diameter (DD) or more, and PC was performed for microaneurysm (MA) when ME relapsed more than 1 year after initiation of IVR.

Previously, we investigated treatment outcomes of BRVO-ME [10]. In that study, the patients initially treated with IVR were divided into healed and refractory cases. Patients with resolution of ME in less than 2 years after the first IVR and no relapse for more than 6 months after the last anti-VEGF injection or those with residual mild ME without visual acuity loss for more than 6 months after the final anti-VEGF injection were classified as healed. Patients who had persistent or relapsed ME within 6 months after receiving anti-VEGF injection for more than 2 years following the initial IVR were classified as refractory. In this study, patients who were healed according to the previous definition as well as patients who were refractory and followed for more than 2 years were classified in the adherence group, and those who were refractory but followed for only less than 2 years were classified in the dropout group.

We compared the demographics as well as baseline BCVA and foveal thickness (FT) between the two groups and the changes in BCVA over the course of treatment in the two groups.

Visual acuity was measured using decimal visual acuity chart and converted to logarithm of the minimal angle resolution (logMAR) for statistical analysis. FT was measured using Zeiss Cirrus OCT (Carl Zeiss Meditec, Inc.) or DRI OCT Triton (Topcon, Inc.). Distance from home to hospital was measured using maps of Geospatial Information Authority of Japan (GSI) (GSI website: https://maps.gsi.go.jp) based on the distance between the patient's address obtained from the medical record and the hospital. All statistical analyses were performed using IBM SPSS Statistics for Windows, version 28.0. Data normality was analyzed using the Shapiro–Wilk test, and the data were found to be not normally distributed. For comparison between the adherence and dropout groups, qualitative variables were analyzed using the chi-square test, and quantitative variables were analyzed using the Mann–Whitney *U* test, a nonparametric test. We calculated 95% confidence intervals (CIs) and also effect sizes by dividing the standardized test statistic by the square root of the sample size. Wilcoxon signed-rank sum test was used to analyze the changes in baseline visual acuity and foveal thickness within each group. Cases with missing values were excluded from calculation for that parameter. A *p* value less than 0.05 was considered statistically significant.

## 3. Results

The clinical characteristics of the adherence and dropout groups at baseline are listed in Table 1. Of 235 patients, 179 patients (76.2%) were classified in the adherence group and 56 patients (23.8%) were classified in the dropout group. There were no differences between the adherence and dropout groups in age, sex ratio, prevalence of hypertension and diabetes, distance from home to hospital, BCVA, and FT at baseline.

Outcomes of the treatment are shown in Table 2. The mean follow-up periods in the adherence and dropout groups were 23.4 ± 14.2 and 7.1 ± 5.5 months, and the number of intravitreal injections of anti-VEGF drug received up to the last follow-up was 4.0 ± 3.4 and 2.7 ± 1.8, respectively. The clinical outcome of ME after initiation of IVR was as follows: ME resolved in less than 1 year in 113 patients (48.0%) and from 1 year to less than 2 years in 24 patients (10.2%) without recurrence for more than 6 months from the last injection; mild ME remained but no visual acuity loss for more than 6 months in 12 patients (5.1%); and ME recurred repeatedly but follow-up for more than 2 years was possible in 30 patients (12.8%). Among all patients, 63.4% completed treatment with complete or partial ME resolution, and 12.8% continued treatment with recurrent or persistent ME. Hence, 76.2% of all patients were in the adherence group, and 23.8% were in the dropout group. Final visual acuity was significantly better in the adherence group than in the dropout group. Final central foveal retinal thickness was significantly lower in the adherence group. For BCVA at the last visit, the 95% CI was [−0.176, 0.000] and effect size was 0.194. For foveal thickness at the final visit, the 95% CI was [−50.000, −10.000] and effect size was 0.203 (Table 2).

**Figure 1:**
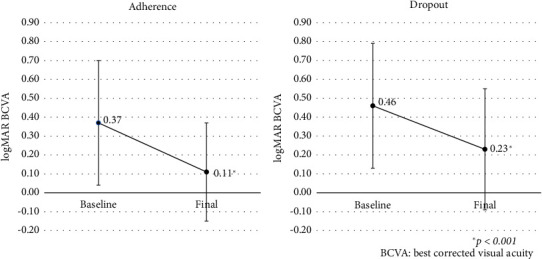
Changes in best-corrected visual acuity.

**Figure 2:**
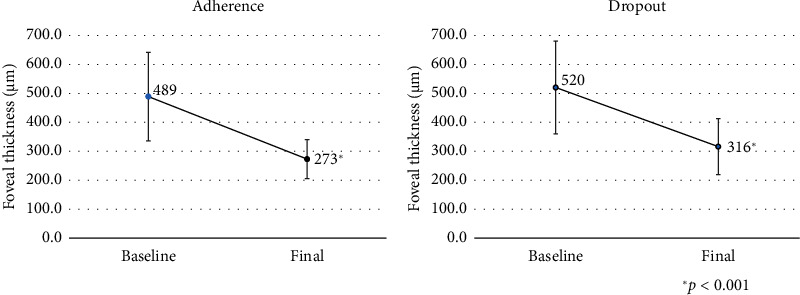
Changes in foveal thickness.

Figure 1 shows the changes in mean BCVA in the adherence and dropout groups. In the adherence group, the mean BCVA was 0.37 before the first IVR injection and 0.11 at the last follow-up, with significant improvement (*p* < 0.001). In the dropout group, the mean BCVA was 0.46 before the first IVR injection and 0.23 at the last follow-up, also with significant improvement (*p* < 0.001).  Figure 2 shows the changes in mean FT in the two groups. In the adherence group, the mean FT was 489 *μ*m before the first IVR injection and 273 *μ*m at the last follow-up, with significant decrease (*p* < 0.001). In the dropout group, the mean FT was 520 *μ*m before the first IVR injection and 316 *μ*m at the last follow-up, also with significant decrease (*p* < 0.001).

The reasons for treatment dropout are shown in Table 3. Reasons for treatment dropout included unknown in 64.3%, patient declining further treatment in 12.5%, progression of dementia in 8.9%, and difficulty in hospital visit due to walking disability in 7.1% of the patients. Other reasons included treatment of other diseases and change of residence.

Analysis of the dropout rates stratified by age group showed relatively high rate of 30.4% in age 50 and under, relatively low rates of 16.3% in the 50s and 19.2% in the 60s, and increasing again to 32.1% in the 70s and 26.4% in age 81 and over.

## 4. Discussion

In this study, the dropout rate of cases initially treated with IVR was 23.8%. Although direct comparison with past study results is not appropriate due to different definitions of dropout, previously reported nonadherence rate for BRVO-ME treatment was 25% [7] and loss to follow-up rate for RVO-ME was 25.4% [9].

No differences in baseline factors including demographics, comorbidities, visual acuity, foveal thickness, and distance from home to hospital were found between patients who dropped out of IVR therapy and those who did not. A previous study also reported no differences in baseline factors identified as risk factors for treatment discontinuation in BRVO-ME [7]. However, longer distance from home to hospital and lower baseline visual acuity were reported to be risk factors for treatment discontinuation in RVO-ME [9]. Long distance from home to the hospital was not a risk factor for dropout in the present study. This may be due to the fact that our hospital is located in the metropolitan area of Japan, whereas the studies that reported distance as a risk factor for discontinuation recruited patients at multiple centers in the United States. Although we did not survey the time taken for patients to travel to the hospital, public transport is well developed in urban areas of Japan, especially Tokyo, providing easy access from various parts of the country. Moreover, workers in offices near our hospital have easy access to our hospital from their workplace. Since there are many work facilities in the neighborhood, the distance from workplace to the hospital may not be a physical or psychological barrier for the workers in the district.

There were potential selection biases in the present study. Patients who presented at our hospital were referred by their local primary care physicians. Assuming that some patients referred by their primary care physicians subsequently did not or could not visit our hospital, the patient background of our subjects may have differed from that of the general patient population. It is possible that patients with potentially low adherence due to intellectual inability to understand the disease or due to physical conditions such as systemic diseases and locomotive syndrome declined referral when being informed of the need to receive treatment at a tertiary care facility and hence were not included in the subject population.

The dropout rates stratified by age showed relatively low rates in the 51–70 age groups. This result is similar to a previous finding that non-young, non-elderly age is a risk-reducing factor in RVO-ME [9]. Patients aged 50 years or younger tended to drop out without unknown cause, while those aged 71 years or older were more likely to drop out for physical reasons (dementia, difficulty of visiting the hospital due to walking disability or physical weakness, and treatment of other diseases). As mentioned in the previous section, the convenience of transportation in urban areas is expected to lower the physical and psychological barriers to hospital visits. Despite this, the fact that treatment dropout for unknown reason was still more common among younger patients suggests that, in addition to the busy work schedule of the working-age population, dropout due to financial problems, dissatisfaction with treatment, and other reasons that patients are reluctant to disclose to their health care providers may be more common among younger patients than other generations. In addition, the living environment may also affect adherence in older patients, such as whether or not they have family members to accompany them to the hospital. In a previous report, “physical problems” were cited as a cause of loss to follow-up in patients on anti-VEGF therapy for RVO-ME, and when AMD and DME were also included, “high cost of the drug” and “no improvement in vision after treatment” were cited [8]. For the medical insurance system in Japan, in principle, patient's copayment rate is 30% for those under 70 years of age and those over 75 years with income comparable to the working population; for patients with lower income, the rate is 20% for those aged 70–74 years and 10% for those over 75 years [11]. The dropout rate did not tend to increase in the age groups that have to pay higher out-of-pocket expenses for treatment. Although the income status of each patient was not investigated, Gao et al. [9] found no significant difference in the treatment discontinuation rate depending on income, while the rate was significantly higher in the uninsured group.

In the present study, the dropout group had worse final visual acuity and greater final central foveal retinal thickness. Elken et al. [7] reported that DME patients with poor adherence to hospital visits had a lower rate of visual acuity improvement and a higher rate of visual acuity loss compared to patients with good adherence, while adherence status did not correlate with visual outcome in BRVO patients. The dropout group in our study had poorer outcome because mild cases that healed after a short period of treatment were included in the adherence group (treatment completion). In addition, while Elken et al. [7] evaluated patients after 1 year of treatment, we extended the observation period to 2 years after the first injection. Patients who continued treatment until ME disappeared after 1 to 2 years and patients who continued treatment for more than 2 years if ME was not completely resolved after 2 years were included in the adherence group. This may be the reason for the superior outcome results in the adherence group, since treatment was continued for a longer period of time in patients who needed treatment. Given the more favorable outcome in terms of visual acuity and foveal retinal thickness in the adherence group, we may be able to utilize these data to encourage adherence by explaining to patients at the beginning of treatment that continuous adherence to treatment until macula edema is resolved may be beneficial for visual acuity.

Limitations of this study include those inherent of retrospective case studies, such as missing information as well as selection and recall biases. Selection biases have been discussed above. Furthermore, the subjects of this study consisted of a relatively small number of patients (235 eyes of 235 patients) treated at a single referral center. Therefore, the results obtained in this study may not be generalizable to the whole population in Japan. We calculated 95% CI and effect sizes. The results suggest that although the study had statistical power to detect significant differences for the two primary outcomes, a larger sample size would have been more reliable. Another limitation is that the reasons for dropout were extracted from the medical records only. We were not able to obtain more detailed information from the subjects when the reason for discontinuation recorded by the physicians was “unknown” or “patient did not wish to continue active treatment.” Hence, possible reasons of dropout could only be inferred from the literature. Especially, among younger patients, there were many cases of sudden discontinuation of visits without giving a reason. It is necessary to assume that there may be hidden reasons, particularly negative factors such as financial problems and dissatisfaction with treatment, that the patients are reluctant to disclose to their doctors.

## 5. Conclusions

The dropout rate of anti-VEGF therapy for BRVO-ME was 23.8%. A common reason for dropout was aggravating physical problems with age. The final visual outcome was poor in the dropout group.

## Figures and Tables

**Table 1:** Baseline characteristics of the patients.

	Adherence	Dropout	*p* value
Number of patients/eyes Percent	179/179 76.2%	56/56 23.8%	

Age, years; mean ± SD (Range)	66.6 ± 11.9 (29–89)	68.7 ± 13.3 (31–89)	0.167

Men/women	69/110	24/32	0.565

Hypertension yes/no	120/58 Unknown 1	39/17	0.755

Diabetes yes/no	12/166 Unknown 1	6/50	0.331

Distance from home to hospital (km) (Range)	10.8 ± 14.9 (0.5–143.6)	8.9 ± 9.8 (0.8–45.2)	0.501

BCVA at baseline, logMAR (range, decimal)	0.37 ± 0.33 (0.02–1.0)	0.46 ± 0.33 (0.04–1.0)	0.057

Foveal thickness at baseline (*μ*m); mean ± SD (*μ*m) (Range)	489 ± 153 (223–1148)	520 ± 160 (222–1112)	0.122

SD: standard deviation; BCVA: best-corrected visual acuity.

**Table 2:** Results of the treatment.

	Adherence	Dropout	*p* value	Effect size	95% CI
Follow-up period, months; mean ± SD (Range)	23.4 ± 14.2 (6–77)	7.1 ± 5.5 (0.03–23)	<0.001	−0.596	[11.00, 17.00]

Total IVI times of anti-VEGF drug; mean ± SD (Range)	4.0 ± 3.4 (1–19)	2.7 ± 1.8 (1–12)	0.031	−0.141	[0.000, 1.000]

Additional treatment, eyes					
Photocoagulation	77	13			
STTA	3	0			
Switching to IVA	23	6			
Outcome of macular edema, eyes					
Disappeared within 1 year	113 (48.1%)	—			
Disappeared in 1-2 years	24 (10.2%)	—			
Mild edema remained for more than 1 year	12 (5.1%)	—			
Recurrence, follow-up possible for over 2 years	30 (12.8%)	—			
None of the above	—	56(23.8%)			

BCVA at final visit, logMAR (Range, decimal)	0.11 ± 0.26 (0.1–1.5)	0.23 ± 0.32 (0.1–1.2)	0.003	0.194	[−0.176, 0.000]

Foveal thickness at final visit, (*μ*m) (Range)	273 ± 67 (189–720) No data in one eye (cause unknown)	316 ± 96 (162–552) No data in one eye (cause unknown)	0.002	0.203	[−50.000, −10.000]

SD: standard deviation; IVI: intravitreal injection; VEGF: vascular endothelial growth factor; STTA: sub-Tenon's triamcinolone acetonide; IVA: intravitreal injection of aflibercept; BCVA: best-corrected visual acuity; CI: confidence interval.

**Table 3:** Reasons for dropping out of treatment stratified by age group.

Age, years	50 and under	51–60	61–70	71–80	81 and over	Total
Number of patients	23	49	73	56	34	235
Dropout, number (percent)	7 (30.4%)	8 (16.3%)	14 (19.2%)	18 (32.1%)	9 (26.4%)	56 (23.8%)
Reason						
Unknown	6	5	10	12	3	36 (64.3%)
Patient declining further treatment	1	2	3		1	7 (12.5%)
Progression of dementia			1	2	2	5 (8.9%)
Difficult to attend hospital (walking disability, etc.)				1	3	4 (7.1%)
Treatment of other diseases				3		3 (5.4%)
Change of residence		1				1 (1.8%)

## Data Availability

The datasets collected and/or analyzed during the current study are available from the corresponding author on reasonable request.
